# Multi-Institutional Validation of Two-Streamed Deep Learning Method for Automated Delineation of Esophageal Gross Tumor Volume Using Planning CT and FDG-PET/CT

**DOI:** 10.3389/fonc.2021.785788

**Published:** 2022-01-24

**Authors:** Xianghua Ye, Dazhou Guo, Chen-Kan Tseng, Jia Ge, Tsung-Min Hung, Ping-Ching Pai, Yanping Ren, Lu Zheng, Xinli Zhu, Ling Peng, Ying Chen, Xiaohua Chen, Chen-Yu Chou, Danni Chen, Jiaze Yu, Yuzhen Chen, Feiran Jiao, Yi Xin, Lingyun Huang, Guotong Xie, Jing Xiao, Le Lu, Senxiang Yan, Dakai Jin, Tsung-Ying Ho

**Affiliations:** ^1^ Department of Radiation Oncology, The First Affiliated Hospital Zhejiang University, Hangzhou, China; ^2^ PAII Inc., Bethesda, MD, United States; ^3^ Department of Radiation Oncology, Chang Gung Memorial Hospital, Linkou, Taiwan; ^4^ Department of Radiation Oncology, Huadong Hospital Affiliated to Fudan University, Shanghai, China; ^5^ Department of Radiation Oncology, Lihuili Hospital, Ningbo Medical Center, Ningbo, China; ^6^ Department of Respiratory Disease, Zhejiang Provincial People’s Hospital, Hangzhou, China; ^7^ Department of Radiation Oncology, The First Hospital of Lanzhou University, Lanzhou, China; ^8^ Department of Radiation Oncology, Haining People’s Hospital, Jiaxing, China; ^9^ Department of Nuclear Medicine, Chang Gung Memorial Hospital, Linkou, Taiwan; ^10^ Independent Researcher, Silver Spring, MD, United States; ^11^ Ping An Technology, Shenzhen, China

**Keywords:** deep learning, PET/CT (18)F-FDG, radiotherapy, segmentation, delineation, esophageal cancer

## Abstract

**Background:**

The current clinical workflow for esophageal gross tumor volume (GTV) contouring relies on manual delineation with high labor costs and inter-user variability.

**Purpose:**

To validate the clinical applicability of a deep learning multimodality esophageal GTV contouring model, developed at one institution whereas tested at multiple institutions.

**Materials and Methods:**

We collected 606 patients with esophageal cancer retrospectively from four institutions. Among them, 252 patients from institution 1 contained both a treatment planning CT (pCT) and a pair of diagnostic FDG-PET/CT; 354 patients from three other institutions had only pCT scans under different staging protocols or lacking PET scanners. A two-streamed deep learning model for GTV segmentation was developed using pCT and PET/CT scans of a subset (148 patients) from institution 1. This built model had the flexibility of segmenting GTVs *via* only pCT or pCT+PET/CT combined when available. For independent evaluation, the remaining 104 patients from institution 1 behaved as an unseen internal testing, and 354 patients from the other three institutions were used for external testing. Degrees of manual revision were further evaluated by human experts to assess the contour-editing effort. Furthermore, the deep model’s performance was compared against four radiation oncologists in a multi-user study using 20 randomly chosen external patients. Contouring accuracy and time were recorded for the pre- and post-deep learning-assisted delineation process.

## Introduction

Gross tumor volume (GTV) contouring is an essential task in radiotherapy planning. GTV refers to the demonstrable gross tumor region. Accurate contouring improves patient prognosis and serves as the basis for further clinical target volume delineation (1). For precise GTV delineation, radiation oncologists often need to consider multimodality imaging of MRI, FDG-PET, contrast-enhanced CT, and radiology reports and other relevant clinical information. This manual process is both labor-intensive and highly variable.

For esophageal cancer, neoadjuvant concurrent chemoradiation therapy is the recommended primary treatment for locally advanced disease in our institution, as relatively fewer patients are first diagnosed at asymptomatic early stages eligible for esophagostomy. Compared to other types of cancers, esophageal GTV contouring has its unique challenges: 1) The esophagus possesses a long cranial to caudal anatomical range, where tumors may appear at any location along this tubular organ. Multifocal tumors are also not uncommon (2, 3). Accurately identifying the tumor location needs significant efforts and expertise from radiation oncologists by referring to multiple examinations, such as panendoscopy, contrast esophagography, or FDG-PET/CT. 2) Assessing the longitudinal esophageal tumor extension is difficult on CT, even with additional information from PET. This leads to considerable GTV contouring variations at the cranial-caudal border (4, 5). 3) Treatment planning CT (pCT) exhibits poor contrast between the esophageal tumor and surrounding tissues. This limitation is addressed by frequently manually referring to adjacent slices to delineate GTV’s radial borders, further increasing the manual burden and time. Therefore, current manual esophageal GTV contouring is labor-intensive and requires extensive experiences of radiation oncologists, otherwise leading to inconsistent delineation. Accurate and automated GTV contouring is of evidently great benefits.

Deep learning methods have been demonstrated as potentially clinically relevant and useful tools in many medical image analysis tasks (6–10). The deep learning-based target volume and organ at risk contouring were also increasingly studied recently (11–17). Nevertheless, for esophageal GTV, the clinical applicability of deep learning-based auto-contouring is still unclear under a multi-institutional evaluation setup.

In this study, we developed and validated a two-streamed three-dimensional (3D) deep learning esophageal GTV segmentation model, which had the flexibility to segment the GTV using only pCT or pCT and PET/CT combined when available. The deep model was developed using 148 patients with pCT and PET/CT imaging from institution 1 and independently validated using 104 unseen patients from institution 1 and 354 patients from three external institutions. Furthermore, using 20 randomly selected patients from external institutions, the deep model performance was compared under a multi-user setting with four board-certified radiation oncologists experienced in esophageal cancer treatment.

## Materials and Methods

### Datasets

**Figure 1:**
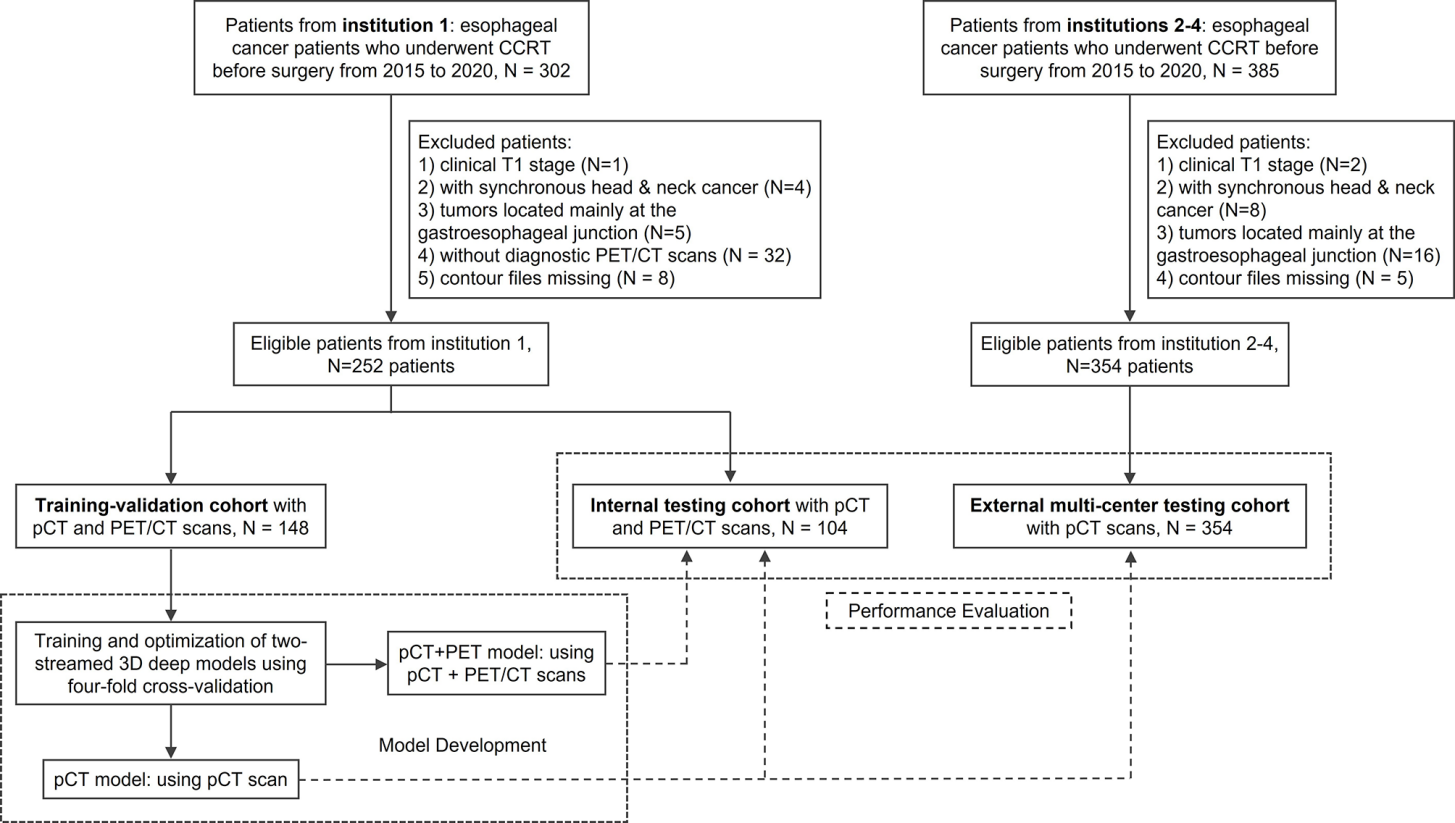
Study flow diagram of esophageal gross tumor volume (GTV) segmentation in a multi-institutional setup. CCRT, concurrent chemoradiation therapy; pCT, treatment planning CT.

A total of 606 patients with esophageal cancer from four institutions were collected in this retrospective study under each institutional review board approval. Requirements to obtain informed consent were waived. All patients had undergone concurrent chemoradiation therapy before surgery between 2015 and 2020. The exclusion criteria are shown in **Figure 1**. All patients had available pCT scans and the corresponding manual GTV contours used for clinical treatment. According to the availability of PET scanner and the staging protocol of different institutions, patients from institution 1 (252 patients total) received additional diagnostic FDG-PET/CT scan, whereas 354 patients from other institutions collected only pCT. Imaging details are described in **Appendix**. A subset of 148 patients from institution 1 was used as the training/validation cohort, while the remaining 104 patients from institution 1 and 354 patients from the other three institutions were treated as unseen internal and external testing cohorts, respectively (**Figure 1**). One hundred forty-eight (institution 1) of the 606 patients were previously reported (18). This prior work dealt with segmentation method developments, whereas in this article, we constructed the deep model using a different implementation (**Appendix**) and evaluated the performance on 458 unseen multi-institutional patients (104 from institution 1; 354 from the other three institutions).

### Model Development

**Figure 2:**
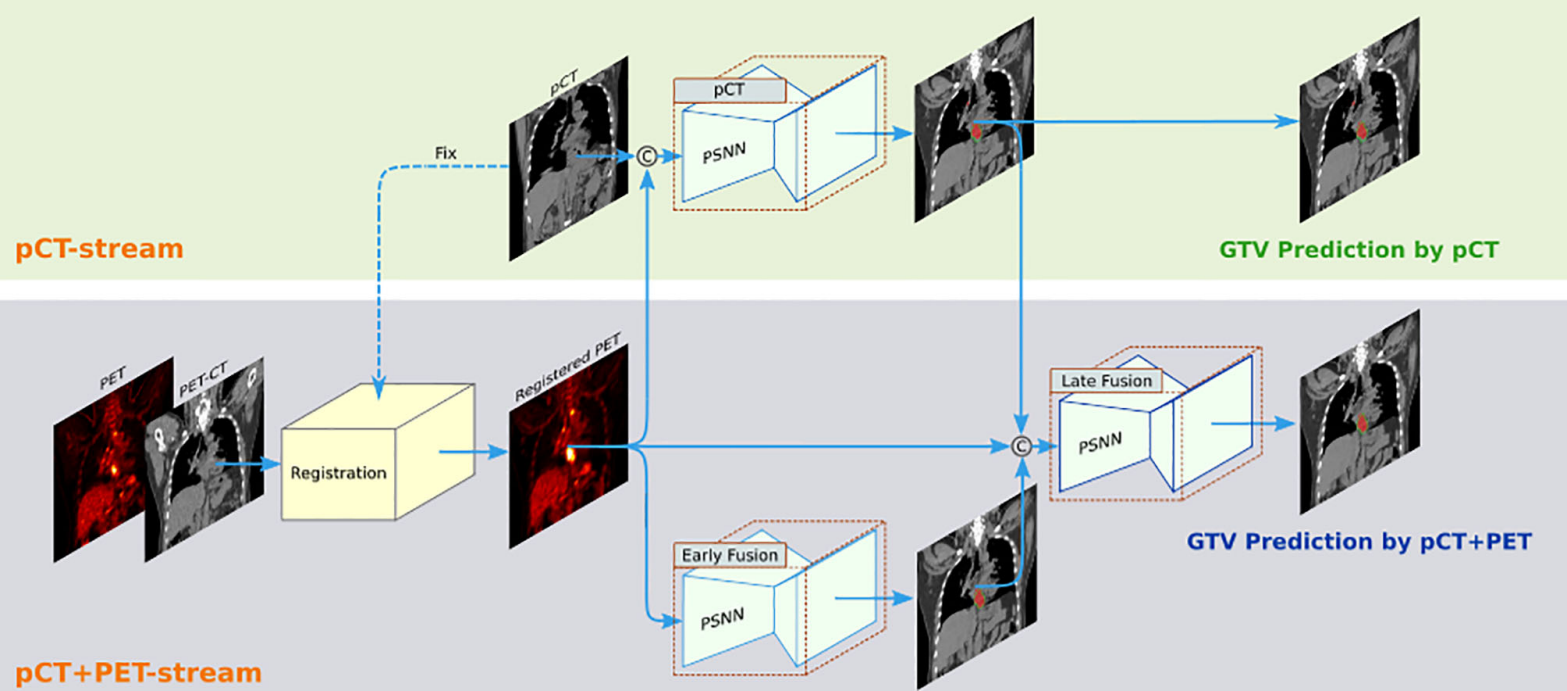
The two-streamed 3D deep learning model for esophageal gross tumor volume (GTV) segmentation using treatment planning CT (pCT) and FDG-PET/CT scans. pCT stream takes the pCT as input and produces the GTV segmentation prediction. The pCT+PET stream takes both pCT and PET/CT scans as input. It first aligns the PET to pCT by registering diagnostic CT (accompanying PET scan) to pCT and applying the deformation field to further map PET to pCT. Then, it uses an early fusion module followed by a late fusion module to segment the esophageal GTV using the complementary information in pCT and PET. This workflow can accommodate to the availability of PET scans in different institutions. Although 3D inputs are used, we depict 2D images for clarity.

We implemented a two-streamed 3D esophageal GTV segmentation method based on the process described in Jin et al. (18, 19), which consisted of a pCT stream to segment GTVs using only pCT input (denoted pCT model) and a pCT+PET stream using an early fusion module followed by a late fusion module to segment GTVs leveraging the joint information in pCT and PET multimodalities (denoted pCT+PET model). The overall segmentation flowchart is illustrated in **Figure 2**. In the pCT+PET stream, PET was aligned to pCT by first registering the diagnostic CT (accompanying the PET) to pCT and applying the deformation field to map PET to pCT. For segmentation backbone, 3D progressive semantically nested network (18) was adopted. Details of the registration, two-streamed formulation, and network architecture are included in **Appendix**.

To obtain the final models for testing, we conducted 4-fold cross-validation (split at the patient level) on the 148 training-validation patients from institution 1. Thereby, 148 patients were randomly partitioned into four equal-size subgroups (25% of patients). Of the four subgroups, a single subgroup was retained as the validation data for model selection, while the remaining three subgroups were used for training. The cross-validation process was repeated four times/4-fold, with each of the four subgroups used once as the validation data. Finally, four deep models were obtained from the four rounds of training. They were ensembled to predict the final GTV contours in all the unseen testing data.

### Quantitative Evaluation of Contour Accuracy

The contouring accuracy was quantitatively evaluated using three common segmentation metrics (11, 12), i.e., Dice similarity coefficient (DSC), 95% Hausdorff distance (HD95), and average surface distance (ASD). For the internal testing, the performance of pCT or pCT+PET model was separately computed. During external testing, the pCT model performance was reported. We also explored the comparison of these metrics in subgroups with different characteristics: clinical T stages and different tumor locations [cervical and upper, middle, and lower third of esophagus according to Japan Esophageal Society (20)].

Additionally, the performance of our two-streamed models was compared with the previous state-of-the-art method (21) using a 3D denseUnet (22, 23) for pCT-based esophageal GTV segmentation. For the model development of Yousefi et al. (21), the same 4-fold cross-validation protocol was applied to ensure a neutral comparison.

### Human Experts’ Assessment of Contour Accuracy

An assessment experiment by human experts was further conducted to evaluate the contour editing efforts required for deep model predictions to be clinically accepted. Specifically, deep learning predictions of 354 patients from three external multi-institutions were distributed to two experts (both >15 years of experience) to assess the degree of manual revision that was defined as the percentage of GTV slices that needed modification for clinical acceptance. Five categories were designated as no revision required, revision required in <10% slices, revision required in 10%–30% slices, revision required in 30%–60% slices, and unacceptable (revision required in >60% slices or prediction completely missed the tumor). We analyzed the correlations between different quantitative metrics and degrees of manual revision.

Note that esophageal GTV may appear at any esophageal location spanning an extensive longitudinal range, which is different from the more spatially constrained anatomical location such as head and neck or prostate (11, 12). Hence, automated esophageal GTV segmentation may identify wrong tumor locations. These scenarios could lead to large or undefined distance errors. Therefore, for the quantitative evaluation, we additionally report the number of patients identified as unacceptable by clinical experts and calculated the DSC, HD95, and ASD metrics using the remaining patients.

### Multi-User Evaluation

We further conducted a multi-user study involving four board-certified radiation oncologists (3–6 years’ experience in treating esophageal cancer) from 4 different institutions. First, pCT of 20 randomly selected patients in the external testing cohort along with their clinical, radiological, and panendoscopy reports and any other useful information were extracted and provided to these four radiation oncologists for manual contouring. Next, after a minimum interval of 1 month, deep model-predicted GTV contours were distributed to these four radiation oncologists for editing along with previously available information. All radiation oncologists were blinded to the ground truth contours and their first-time contours. Accuracy of our deep model predictions was compared to the multi-user performance in terms of DSC, HD95, and ASD. Similar to Lin et al. (11), interobserver variations were assessed using multi-user DSC and volume coefficient of variation (the ratio between standard deviation and mean). Times used for the pre- and post-deep learning-assisted contouring were recorded.

### Statistical Analysis

The Wilcoxon matched-pairs signed rank test was used to compare 1) DSC, HD95, and ASD scores between the pCT model and pCT+PET model in the internal testing set and between the proposed model and 3D DenseUNet method in the external testing set; 2) DSC, HD95, ASD, and time taken of pre- *vs.* post-deep learning-assisted contouring in multi-user evaluation. Mann–Whitney U test was used to compare DSC, HD95, and ASD at different clinical T stages. Multiple linear regression with stepwise model selection was used to compare the metrics at different tumor locations, since a large tumor may locate across multiple esophagus regions. Spearman correlation coefficients were assessed for mean DSC, HD95, and ASD vs. degrees of manual revision, respectively. The *χ* ^2^ test was used to compare the difference in degrees of manual revision between subgroups. All analyses were performed by using R (24). Statistical significance was set at two-tailed *p* < 0.05.

## Results

A total of 606 esophageal cancer patients were included. **Table 1** summarizes the main characteristics of the entire cohort, and the separated training-validation, internal testing, and external testing cohorts. Characteristics of the 20 randomly selected patients used in multi-user evaluation are presented in Appendix Table A1.

**Table 1:** Subject and imaging characteristics.

Characteristics	Entire cohort Institutions 1–4(n = 606)	Training-validation Institution 1(n = 148)	Internal testing Institution 1(n = 104)	External testing Institutions 2–4(n = 354)
Sex	…	…	…	…
Male	537 (89%)	135 (91%)	98 (94%)	304 (86%)
Female	69 (11%)	13 (9%)	6 (6%)	50 (14%)
Diagnostic age	65 [57–72]	55 [50–61]	56 [50–62]	67 [61–75]
Clinical T stage	…	…	…	…
cT2	116 (19%)	24 (16%)	18 (17%)	74 (21%)
cT3	306 (51%)	71 (48%)	58 (56%)	177 (50%)
cT4	184 (30%)	53 (36%)	28 (27%)	103 (29%)
Tumor location	…	…	…	…
Cervical	81 (13%)	11 (7%)	10 (10%)	60 (17%)
Upper third	204 (34%)	26 (18%)	35 (34%)	143 (40%)
Middle third	325 (54%)	84 (57%)	63 (61%)	178 (50%)
Lower third	174 (29%)	69 (47%)	35 (34%)	70 (20%)
BMI	…	…	…	…
<18.5	121 (20%)	22 (15%)	15 (14%)	84 (24%)
18.5–23.9	393 (65%)	94 (63%)	59 (57%)	240 (68%)
>24	92 (15%)	32 (22%)	30 (29%)	30 (8%)
Imaging available	…	…	…	…
pCT	606 (100%)	148 (100%)	104 (100%)	354 (100%)
PET/CT	252 (42%)	148 (100%)	104 (100%)	0 (0%)

Patients may have tumors located across multiple esophagus regions; hence, total numbers summed at various tumor locations for the entire and sub-institution cohorts are greater than the corresponding total patient numbers. Age is presented as median and [interquartile range].

cT2, clinical T stage 2; cT3, clinical T stage 3; cT4, clinical T stage 4; BMI, body mass index; pCT, treatment planning CT.

### Performance in the Internal Testing Set

Quantitative performance of our deep model in the internal testing set is summarized in **Tables 2**, **3**. For the pCT model, we observed the mean and 95% confidence interval of DSC, HD95, and ASD as 0.81 (0.79, 0.83), 11.5 (9.2, 13.7) mm, and 2.7 (2.2, 3.3) mm, respectively. In the subgroup analysis (**Appendix Figures A3, A4**), the pCT model achieved a significantly higher mean DSC for advanced T stage patients (cT3, cT4) than those in the early cT2 patients (0.82 and 0.82 vs. 0.76, *p* < 0.05). The tumor locations exhibited no significant performance differences. With additional PET scans, the pCT+PET model significantly increased the performance to 0.83 (0.81, 0.84), 9.5 (8.0, 10.9) mm, and 2.2 (1.9, 2.5) mm with *p* < 0.01 in DSC, HD95, and ASD, respectively. **Figure 4** **A** shows several qualitative examples for GTV segmentation in the internal testing set.

**Table 2:** Quantitative results of esophageal GTV segmentation by the pCT model in the unseen internal testing data.

	Institution 1 (Unseen Internal Testing) Using pCT Model			
	Unacceptable Number (percentage)	DSC mean (95% CI)	HD95 (mm) Mean (95% CI)	ASD (mm) Mean (95% CI)
Total patients (n = 104)	8 (8%)	0.81 (0.79, 0.83)	11.5 (9.2, 13.7)	2.7 (2.2, 3.3)
Clinical T stage				
cT2 (n = 18)	4 (22%)	0.76 (0.67, 0.86)	12.0 (5.5, 18.4)	3.0 (1.0, 5.1)
cT3 (n = 58)	3 (5%)	0.82 (0.80, 0.84)	10.7 (7.9, 13.5)	2.5 (1.9, 3.2)
cT4 (n = 28)	1 (4%)	0.82 (0.79, 0.85)	12.8 (7.9, 17.7)	3.0 (2.0, 4.0)
Tumor location				
Cervical (n = 10)	1 (10%)	0.82 (0.75, 0.89)	9.2 (6.5, 12.0)	2.2 (1.5, 2.8)
Upper third (n = 35)	1 (3%)	0.83 (0.81, 0.85)	9.6 (7.4, 11.9)	2.2 (1.8, 2.5)
Middle third (n = 63)	5 (8%)	0.80 (0.78, 0.83)	12.0 (8.9, 15.0)	2.9 (2.2, 3.6)
Lower third (n = 35)	2 (6%)	0.81 (0.77, 0.85)	13.3 (8.6, 18.0)	3.3 (2.1, 4.5)

GTV, gross tumor volume; CI, confidence interval; DSC, Dice similarity coefficient; HD95, 95% Hausdorff distance; ASD, average surface distance; cT2, clinical T stage 2; cT3, clinical T stage 3; cT4, clinical T stage 4; pCT, treatment planning CT.

**Table 3:** Quantitative results of esophageal GTV segmentation by the pCT+PET model in the unseen internal testing data.

	Institution 1 (Unseen Internal Testing) Using pCT+PET Model			
	Unacceptable Number (percentage)	DSC Mean (95% CI)	HD95 (mm) Mean (95% CI)	ASD (mm) Mean (95% CI)
Total patients (n = 104)	4 (4%)	0.83 (0.81, 0.84)	9.5 (8.0, 10.9)	2.2 (1.9, 2.5)
Clinical T stage				
cT2 (n = 18)	3 (17%)	0.77 (0.69, 0.85)	11.4 (6.3, 16.6)	2.7 (1.3, 4.2)
cT3 (n = 58)	0 (0%)	0.84 (0.82, 0.85)	9.0 (7.0, 11.0)	2.0 (1.7, 2.4)
cT4 (n = 28)	1 (4%)	0.84 (0.82, 0.86)	9.3 (7.3, 11.4)	2.3 (1.9, 2.6)
Tumor location				
Cervical (n = 10)	1 (10%)	0.83 (0.78, 0.89)	9.4 (6.2, 12.7)	2.0 (1.5, 2.5)
Upper third (n = 35)	0 (0%)	0.84 (0.82, 0.86)	8.1 (6.2, 10.0)	1.9 (1.6, 2.2)
Middle third (n = 63)	3 (5%)	0.83 (0.81, 0.84)	9.5 (8.0, 11.1)	2.2 (1.9, 2.5)
Lower third (n = 35)	0 (0%)	0.83 (0.79, 0.86)	10.8 (7.5, 14.0)	2.6 (1.9, 3.3)

GTV, gross tumor volume; CI, confidence interval; DSC, Dice similarity coefficient; HD95, 95% Hausdorff distance; ASD, average surface distance; cT2, clinical T stage 2; cT3, clinical T stage 3; cT4, clinical T stage 4; pCT, treatment planning CT.

### Performance in the External Testing Set

In the external multi-institutional testing, we observed the mean and 95% CI of DSC, HD95, and ASD as 0.80 (0.78, 0.81), 11.8 (10.1, 13.4) mm, and 2.8 (2.4, 3.2) mm, respectively, using the pCT model (**Table 4**). These values did not show significant differences compared to those during the internal testing. Our pCT-based GTV segmentation model generalized well to patients of three other institutions. In the subgroup analysis, a similar trend was observed as internal testing: deep model obtained markedly improved DSC and HD95 in advanced cT3 and cT4 patients vs. early cT2 patients (mean DSC 0.81 and 0.82 vs. 0.71, *p* < 0.001; mean HD95 11.4 and 11.4 mm vs. 13.8 mm, *p* ≤ 0.001).

**Table 4:** Quantitative results of esophageal GTV segmentation by the pCT model in the multi-institutional external testing data.

	Institutions 2–4 (External Multi-Institutional Testing) Using pCT Model			
	Unacceptable Number (percentage)	DSC Mean (95% CI)	HD95 (mm) Mean (95% CI)	ASD (mm) Mean (95% CI)
Total patients (n = 354)	33 (9%)	0.80 (0.78, 0.81)	11.8 (10.1, 13.4)	2.8 (2.4, 3.2)
Clinical T stage				
cT2 (n = 74)	23 (31%)	0.71 (0.66, 0.76)	13.8 (10.0, 17.5)	3.6 (2.5, 4.8)
cT3 (n = 177)	5 (3%)	0.81 (0.80, 0.82)	11.4 (8.8, 13.9)	2.6 (2.1, 3.2)
cT4 (n = 103)	5 (5%)	0.82 (0.80, 0.83)	11.4 (9.3, 13.6)	2.7 (2.1, 3.3)
Tumor location				
Cervical (n = 60)	4 (6%)	0.80 (0.78, 0.82)	11.7 (8.6, 14.8)	2.5 (1.7, 3.3)
Upper third (n = 143)	11 (8%)	0.79 (0.77, 0.81)	12.6 (10.4, 14.9)	3.0 (2.4, 3.7)
Middle third (n = 178)	14 (8%)	0.80 (0.78, 0.81)	11.5 (9.3, 13.5)	2.9 (2.4, 3.5)
Lower third (n = 70)	5 (7%)	0.80 (0.78, 0.82)	15.4 (9.3, 21.5)	3.3 (2.1, 4.5)

GTV, gross tumor volume; CI, confidence interval; DSC, Dice similarity coefficient; HD95, 95% Hausdorff distance; ASD, average surface distance; cT2, clinical T stage 2; cT3, clinical T stage 3; cT4, clinical T stage 4; pCT, treatment planning CT.

When compared with the previous leading 3D DenseUNet (21), its DSC, HD95, and ASD scores were all inferior to our model performance, e.g., mean DSC 0.75 vs. 0.80, *p* < 0.001 (**Appendix Table A2**).

### Human Experts’ Assessment

**Figure 3:**
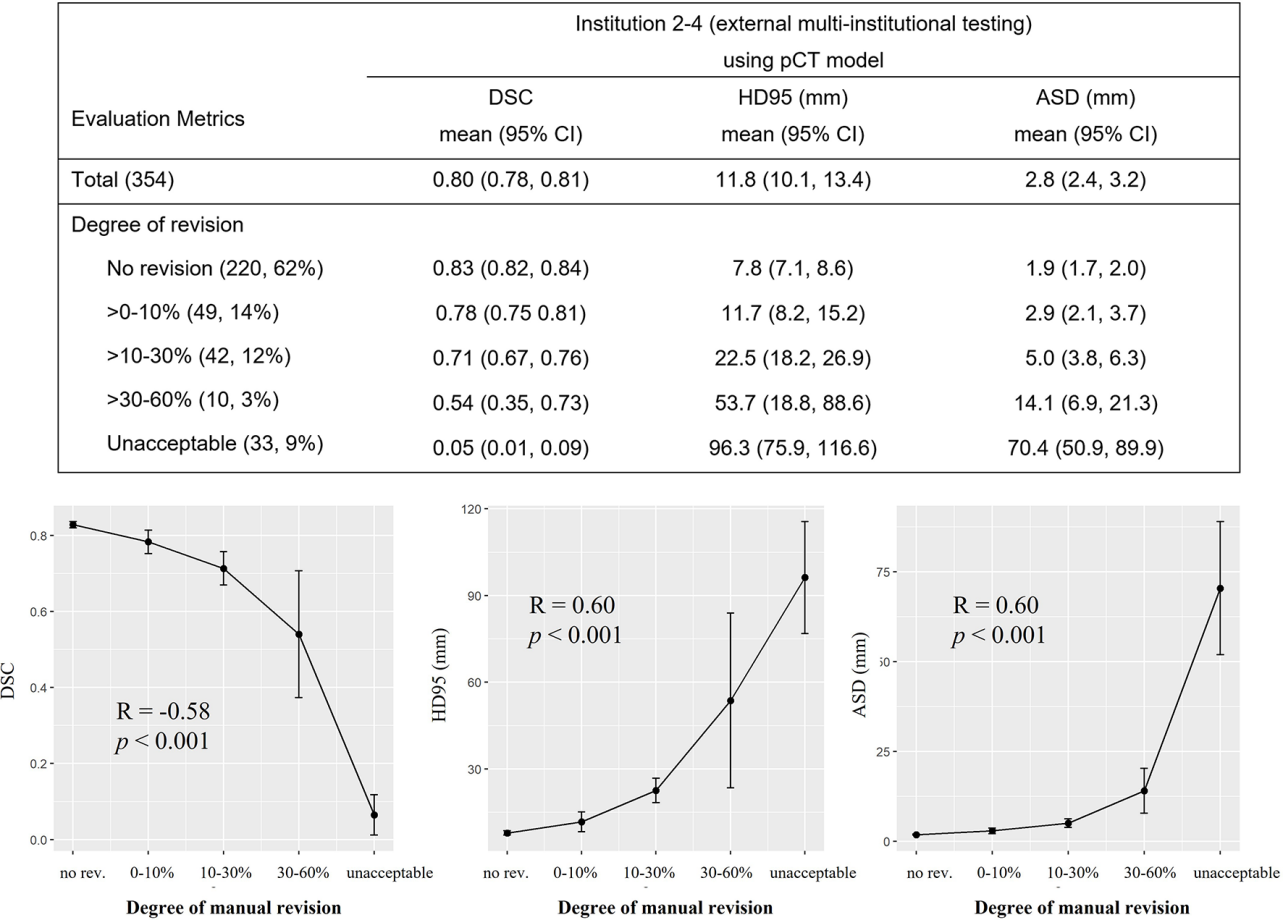
Expert assessment of manual revision degree of the deep model-predicted contours. Table in the top row summarized the mean and 95% confidence interval (CI) of Dice similarity coefficient (DSC), 95% Hausdorff distance (HD95), and average surface distance (ASD) stratified by different degrees of manual revision. The correlations between the mean of DSC, HD95, ASD, and the degree of manual revision were plotted in the bottom row. Spearman correlation coefficient showed that DSC and degree of manual revision were correlated (R = -0.58, *p* < 0.001). Same correlation was observed for the HD95 and ASD (HD95: R = 0.60, *p* < 0.001; ASD: R = 0.60, *p* < 0.001). Degree of manual revision was defined as the percentage of gross tumor volume (GTV) slices that needed modification for clinical acceptance. pCT, treatment planning CT.

**Figure 4:**
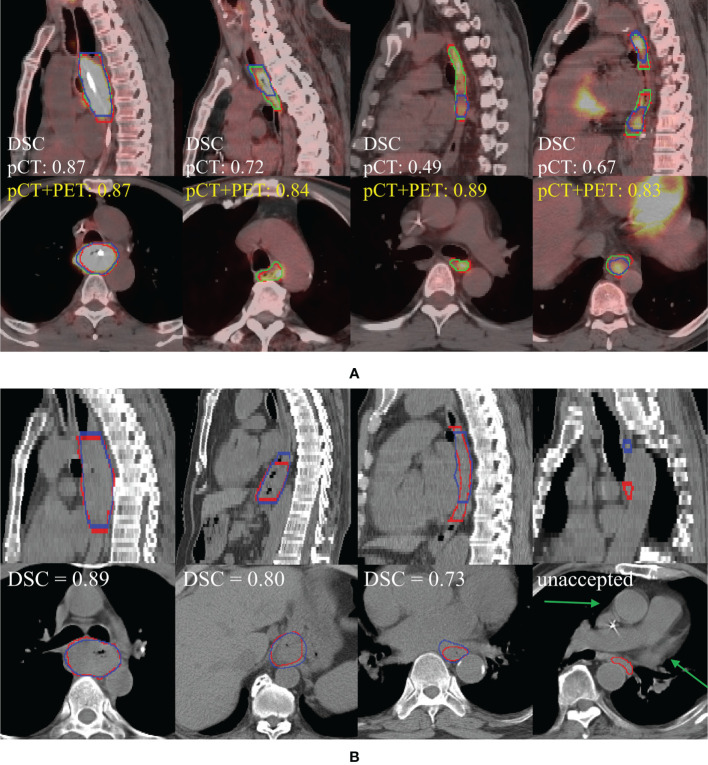
**(A)** Performance comparison of pCT model and pCT+PET in the internal testing set (left to right: cT4, cT3, cT3, multifocal cT2). Red, green, and blue show the contours of ground truth reference, pCT+PET model prediction, and pCT model prediction, respectively. **(B)** Performance examples of pCT model in the external testing set according to the degree of manual revision (left to right): no revision, >0%–30%, >30%–60%, and unacceptable. Red and blue show the contours of ground truth reference and pCT model prediction, respectively. Green arrow points to the uncommon anatomy for the unacceptable case in the rightmost column. pCT, treatment planning CT; DSC, Dice similarity coefficient.

Human experts’ assessment showed that the majority (311 of 354, 88%) of deep learning predictions in the external testing set were clinically accepted or required only minor editing (no revision, n = 220; 0%–30% revision, n = 91). Ten (3%) patients had contouring errors in 30%–60% slices, and 33 (9%) patients had unacceptable predictions that required substantial editing efforts. **Figure 3** details the assessment results. The mean DSC, HD95, and ASD were correlated to the degrees of manual revision (DSC: R = -0.58, *p* < 0.001; HD95: R = 0.60, *p* < 0.001; ASD: R = 0.60, *p* < 0.001). These results indicated the reliability of using DSC, HD95, and ASD as contouring accuracy evaluation criteria, consistent with the contour editing effort necessitated in actual clinical practice. Thirty-three (9%) patients had unacceptable predictions where our model failed to accurately locate the tumor, leading to small dice and large distance errors. Among 33 unaccepted cases, 23 (70%) patients had cT2 tumors. Other cases often exhibited relatively uncommon scanning position or anatomies (see the rightmost column in **Figure 4B**). In the subgroup analysis (**Appendix Table A3**), a significantly smaller percentage of patients required major revision (>30% slice revision) in advanced cT3 and cT4 stages as compared to that in early cT2 stage (5% and 8% vs. 35%, *p* < 0.01). Tumor locations did not significantly impact the degrees of manual revision.

### Multi-User Evaluation

**Figure 5:**
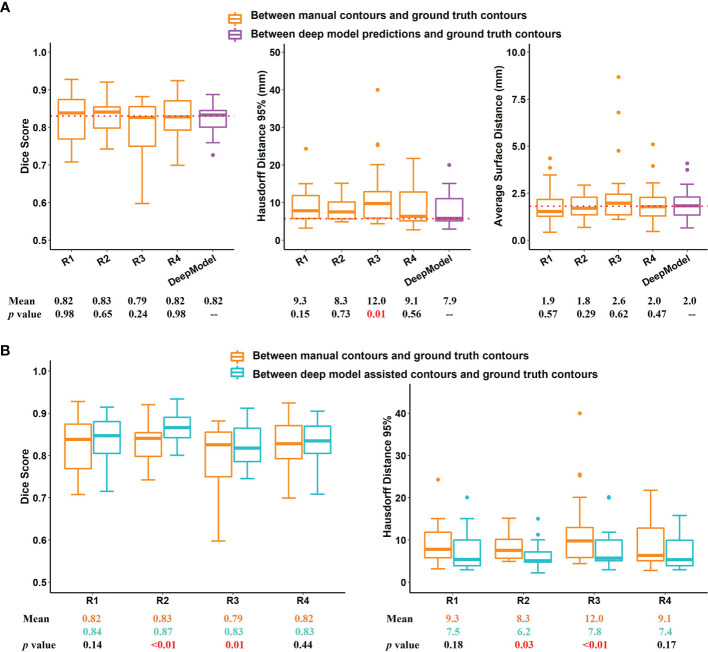
Results of multi-user evaluation. **(A)** Boxplot of Dice similarity coefficient (DSC), 95% Hausdorff distance (HD95), and average surface distance (ASD) for the comparison of manual contours of four radiation oncologists with our treatment planning CT (pCT)-based deep model-predicted contours. Dotted lines indicate the median DSC, HD95, and ASD of our pCT model performance. **(B)** Comparison of DSC and HD95 between second-time deep learning-assisted contours with those of first-time manual contours. R1 to R4 represent the 4 radiation oncologists involved in the multi-user evaluation. DeepModel is our pCT model.

Performance evaluation of our pCT model with four board-certified radiation oncologists is shown in **Figure 5** and **Appendix Table A4**. Among 20 testing cases, our model performed comparably to these four radiation oncologists in terms of DSC and ASD (mean DSC: 0.82 vs. 0.82, 0.83, 0.79, 0.82; mean ASD: 2.0 mm vs. 1.9, 1.8, 2.6, 2.0 mm). For HD95, our model achieved the lowest mean HD95 errors among all results (significantly smaller than R3, mean HD95 7.9 mm vs. 12.0 mm, *p* = 0.01).

**Figure 6:**
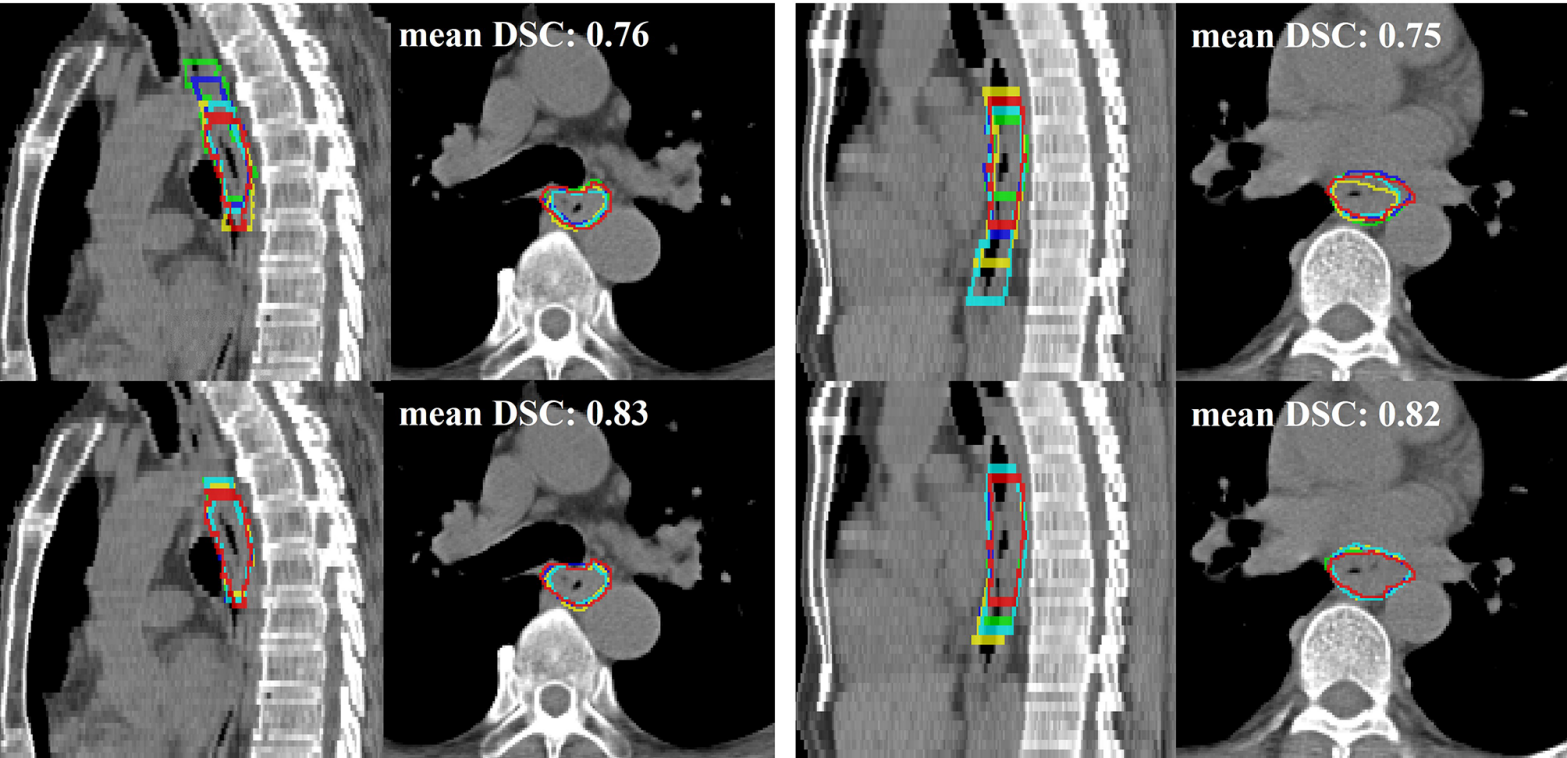
Two qualitative examples (left and right) in sagittal and axial views of comparison between the first-time manual contour (top row) and second-time deep learning-assisted contours (bottom row). Red is the ground truth contour, while green, blue, yellow, and cyan represent the other four radiation oncologists’ contours. The average Dice similarity coefficient (DSC) of 4 radiation oncologists for their first-time manual contour is 0.76 and 0.75 to the two examples, respectively. The DSC performance improved to 0.83 and 0.82 for their second-time contour with assistance from the deep learning predictions.

Next, we examined if the accuracy of manual contouring could be improved with assistance of deep model predictions. It is observed that when editing upon deep model predictions, 2 out of 4 radiation oncologists’ performance had been significantly improved in DSC and HD95 (**Figure 5** and **Appendix Table A5**). The inter-user variation was also reduced (**Figure 6**). Mean multi-user DSC was improved from 0.82 to 0.84 (*p* < 0.001), and mean volume coefficient of variation was reduced by 37.6% (from 0.14 to 0.09, *p* = 0.03). Furthermore, the contouring time had been reduced by an average of 48.0% (from 10.2 to 5.3 min). Our pCT model takes an average of 20 s to predict one patient.

## Discussion

In this multi-institutional study, we developed a two-streamed 3D deep learning model to segment esophageal GTV trained on 148 patients with both treatment planning CT (pCT) and PET/CT scans from institution 1. The performance was extensively evaluated using 104 unseen institution 1 patients and 354 external multi-institutional patients. Our pCT model achieved mean DSC and ASD of 0.81 and 2.7 mm in the internal testing and generalized well to the external testing with mean DSC and ASD of 0.80 and 2.8 mm. Adding PET scans, the pCT+PET model further boosted DSC and ASD to 0.83 and 2.2 mm for the internal testing. From a multi-user study, the pCT model performed favorably when compared against four board-certified radiation oncologists in metrics of DSC and ASD while achieving the smallest HD95 errors. By allowing radiation oncologists to edit the deep model predictions, the overall accuracy was improved, and inter-observer variation and contouring time were reduced by 37.6% and 48.0%, respectively.

Accurate GTV delineation improves patient’s prognosis (1). Manual contouring of esophageal GTV on pCT highly relies on the expertise and experiences of radiation oncologists, leading to substantial inter-user variations (4, 5, 25). In clinical practice, radiation oncologists almost always need to refer to other information such as panendoscopy report to determine the tumor range, which is not trivial, requiring the “virtual fusion” of panendoscopy information with pCT image in their minds. In this context, our deep model could benefit radiation oncologists by improving their contouring accuracy and consistency and reducing time spent.

Previous works showed potential clinical applicability of deep learning for the GTV contouring in head and neck and prostate cancers (11, 12). However, for esophageal cancer, studies often collected limited single-institution data for both training and testing (18, 21, 26). For example, a 73% Dice score was achieved when trained and tested on a total of 49 patients (21). In this work, with our deep model developed using 148 patients from the internal institution 1, we extensively evaluated the GTV segmentation performance using 104 unseen internal patients and 354 external multi-institutional patients. Robust performance generalizability to the external multi-institutional testing data was observed despite variations of CT scanner types, imaging protocols, and patient populations.

Generalizability of deep learning models was often the bottleneck for successful clinical deployment. As shown in Zhang et al. (27), direct deployment of well-trained MRI-based prostate and left arterial segmentation models to the unseen data from different centers led to averaged >10% DSC decrease. Good generalizability of our model may come from the following: 1) relative standardized imaging protocols for pCT from various institutions despite different pCT scanner vendors; 2) physically well-calibrated HU values in CT; 3) the 148 training patients from institution 1 are relatively sufficient for covering different CT characteristics of esophageal tumors; and 4) we have effectively trained our two-streamed deep GTV networks.

The developed two-streamed model has demonstrated the flexibility of segmenting esophageal GTV according to the availability of PET/CT scans. For institutions where PET/CT scans are not included as a standardized staging protocol, our pCT model already achieved high accuracy comparable to the inter-user agreement. When PET/CT scans were available, the pCT+PET model could further improve the performance (mean DSC of pCT vs. pCT+PET: 0.81 vs. 0.83, *p* = 0.01).

This study has a few limitations. First, patients in the external test set do not have PET/CT scans because PET is either not available or not required for esophageal cancer staging in three external institutions. Hence, we have not directly validated the performance of our pCT+PET model in the external data. However, considering that tumor contrast in PET is often prominent and can be assessed as a semiquantitative standard uptake value (SUV), we believe that it would not significantly impact our pCT+PET model performance when applied to external patients. Second, the pCT model obtained lower performance for patients of cT2 as compared to those of advanced clinical T stages. This may be because cT2 tumors often exhibited less prominent imaging features in CT. After adding PET, this phenomenon was less evident. Another potential solution might be combining the panendoscopy report information with a deep learning model, which could be optimized by restricting longitudinal ranges. Third, we excluded patients with the primary tumor at gastroesophageal junction, since they were relatively rare (<2%) in our study population and some were treated by surgery. Further investigation of developing the deep learning model on this subpopulation would be of clinical interest. Lastly, we did not include GTV of lymph nodes (GTV_LN_) and clinical target volume (CTV) that are essential for a comprehensive esophageal cancer target contouring tool in this proposed model. GTV_LN_ is a vital part in treating esophageal cancer. However, in this work, our deep model only includes the main esophageal tumor and focuses on the multi-institutional clinical evaluation of tumor GTV auto-contouring because metastatic lymph node identification is a non-trivial problem itself. For example, detecting and subsequently segmenting the metastatic regional lymph nodes, which may spread to a long longitudinal range along the esophagus, would require the development of dedicated deep learning models (28). Note that we have developed recent state-of-the-art technical solutions along this line of work on finding and segmenting GTV_LN_ (29–31). Nevertheless, GTV_LN_ identification is highly challenging, so further technical improvement is needed to achieve clinically applicable performance. We leave the thorough clinical evaluation of GTV_LN_ auto-contouring as our next step of future work. In addition, CTV is another indispensable volume to be delineated in esophageal cancer radiotherapy. We have developed a deep learning-based technical solution to incorporate the 3D spatial context of tumors and lymph nodes for CTV auto-contouring (32). The current main roadblock is on the auto-contouring of GTV_LN_. Once we solve the lymph node problem, we are ready to implement and conduct a large-scale clinical evaluation on the esophageal CTV auto-contouring task.

To conclude, we developed and clinically validated an effective two-streamed 3D deep model that can reliably segment the esophageal GTV using two protocols of pCT alone or pCT+PET/CT. Predicted GTV contours for 88% of patients were in close agreement with the ground truth and could be implemented and adopted clinically where only minor or no editing efforts are required.

## Data Availability Statement

The data analyzed in this study is subject to the following licenses/restrictions: The dataset may be available upon request and application for the institutional research boards’ approval of each participating institution. Requests to access these datasets should be directed to T-YH, albertyho@gmail.com.

## Ethics Statement

Patients from the four institutions were collected in this retrospective study under each institutional review board approval. Requirements to obtain informed consent were waived.

## Author Contributions

First co-authors: XY helped collect the external data, review and modify contours from external institutions, and coordinated multi-user study. DG was responsible for the data cleaning, deep learning model development, and internal and external evaluation. They both designed the experiments and drafted the article. C-KT, P-CP, and T-MH approved the contours for training and validation of the internal institution. JG, YR, and LZ helped collect and organized the external data. XZ, LP, YC, XC, DC, and JY carried out the contouring experiments before and after assistance of deep learning. C-YC and YZC collected and organized internal data. YX, LH, GX, and JX contributed to the design and implementation of the research. LL interpreted the results and drafted the article. DJ was responsible for conception and design of the experiment, development of the deep learning model, and overseeing the evaluation process. SY was responsible for coordinating the external institutions and reviewing and modifying the contours from external institutions, and he also provided guidance and consulting in multi-user study. FJ conducted the statistical analysis and aided in interpreting the results. DJ and T-YH conceived the study, were in charge of overall direction and planning, and drafted the article.

## Funding

This work is partially supported by Maintenance Project of the Center for Artificial Intelligence in Medicine (Grant CLRPG3H0012, CMRPG3K1091, SMRPG3I0011) at Chang Gung Memorial Hospital.

## Conflict of Interest

DG, LL, and DJ were employed by PAII Inc. YX, LH, GX, and JX are employed by Ping An Technology.

The remaining authors declare that the research was conducted in the absence of any commercial or financial relationships that could be construed as a potential conflict of interest.

## Publisher’s Note

All claims expressed in this article are solely those of the authors and do not necessarily represent those of their affiliated organizations, or those of the publisher, the editors and the reviewers. Any product that may be evaluated in this article, or claim that may be made by its manufacturer, is not guaranteed or endorsed by the publisher.
