# Supplementary material for: Multi-Institutional Validation of Two-Streamed Deep Learning Method for Automated Delineation of Esophageal Gross Tumor Volume Using Planning CT and FDG-PET/CT
Source: Front Oncol. 2022 Jan 24;11:785788. doi: 10.3389/fonc.2021.785788 (PMC8820194; doi:10.3389/fonc.2021.785788)
Supplement: Supplementary file 1 [file DataSheet_1.pdf]

Supplemental Materials and Methods

Imaging protocols:

Planning CT (pCT) images from the 4 institutions were acquired with scanners from various vendors, including Siemens and GE healthcare. Slice thickness and in-plane pixel size ranged from 0.78×0.78×1.25mm to 1.37×1.37×5mm (median resolution 0.98×0.98×5mm). The FDG-PET/CT image pairs of 252 patients in institution 1 were acquired using GE Discovery ST scanner (Milwaukee, WI), or Siemens Biograph mCT PET/CT scanner (Hoffman Estates, IL). Before PET acquisition a diagnostic quality CT was performed with 5 mm slice width and average 1mm in-plane pixel size. The PET images were reconstructed with CT-based attenuation correction and the axial spatial resolutions of PET were 4.80 and 2.16 mm for the Discovery ST and Biograph mCT scanners, respectively.

PET/CT to pCT registration:

Direct PET to pCT registration can lead to large errors because of the completely different modalities (1). To overcome this challenge, we register the PET-CT (accompanying the PET) to pCT and use the resulting deformation field to align the PET to pCT. This intra-patient CT-based registration is a much feasible task, where many deformable registration algorithms have demonstrated excellent results. In this work, we used the Dense Displacement Sampling algorithm (DEEDS) (2), which achieved the leading performance of an average target registration error ≤ 2mm in a pulmonary registration challenge (3).

However, to achieve robust performance, PET-CT and pCT require a reasonable rigid initialization to manage their pose, scanning range, and respiratory differences before applying the deformable algorithm. To accomplish this step of registration, we used 3D mass centers of lungs in two CT scans as the initial matching position, where 3D lung masks were automatically segmented using the method of (4). This led to a reliable initial alignment for the chest and upper abdominal regions, ensuring the success of deformable registration process. The resulting deformation field is subsequently applied to PET scan and aligned it to the respective pCT scan. A registration example is shown in Figure A1.

Two-streamed GTV segmentation workflow:

A two-streamed 3D deep learning model for esophageal GTV segmentation was developed, which had the flexibility to segment the GTV using only pCT, or pCT+PET/CT when PET/CT is available. One pCT-based deep network was trained using the only pCT to segment the GTV (denoted as pCT-stream). The other pCT+PET stream consisted of an early fusion network followed by a late fusion network to segment the GTV, which utilizes the complementary information in pCT and PET scans. More details of this method can be found in (5).

Progressive semantically-nested network (PSNN) architecture:

The detailed network architecture of PSNN is shown in Figure A2. It incorporates the strengths of both UNet (6) and PHNN (7) by using deep supervision (8) to progressively propagate high-level semantic features to lower-levels, but higher resolution features.

Training and inference details:

We used a 3D volume of interest (VOI) patch-based fashion to train all the deep networks in the two-streamed method. To generate the 3D training samples, we extracted 96 ×96 ×64 sub-volumes in two manners. First, to ensure enough VOIs with positive GTV content, we randomly extract VOIs centered within the ground truth GT V masks. Second, to obtain sufficient negative examples, we randomly sample 20 VOIs from the rest of the whole volume. We further applied extensive data augmentation, e.g., horizontal flipping, random rotations in the x-y plane within ±10 degrees, intensity scaling between [0.75, 1.25], Gaussian noise with zero mean and (0, 0.1) variance. The optimizer is stochastic gradient descent with the Polynomial learning rate policy. The initial learning rate is 0.01 and a Nesterov momentum of 0.99. Batch size is set to 12 for all networks. The pCT and early fusion deep network were trained for 150 epochs to convergence, while the late fusion deep network was trained for 50 epochs. For inference, we first cropped out the lung xy ranges using the 3D lung mask, then, 3D sliding windows with sub-volumes of 96 ×96 ×64 and strides of 64 ×64 ×32 voxels were used. The probability maps of sub-volumes were aggregated to obtain the whole volume prediction. We implemented our model using Pytorch and Titan-V GPU.

Supplemental References

1. Mattes D, Haynor DR, Vesselle H, et al. Pet-ct image registration in the chest using free-form deformations. *IEEE transactions on medical imaging* 2003;22:120-128.

2. Heinrich MP, Jenkinson M, Brady M, et al. Mrf-based deformable registration and ventilation estimation of lung ct. *IEEE transactions on medical imaging* 2013;32:1239-1248.

3. Murphy, Keelin, et al. "Evaluation of registration methods on thoracic CT: the EMPIRE10 challenge." *IEEE transactions on medical imaging* 30.11 (2011): 1901-1920.

4. Jin, Dakai, et al. "CT-realistic lung nodule simulation from 3D conditional generative adversarial networks for robust lung segmentation." *International Conference on Medical Image Computing and Computer-Assisted Intervention*. Springer, Cham, 2018.

5. Jin, Dakai, et al. "DeepTarget: Gross tumor and clinical target volume segmentation in esophageal cancer radiotherapy." *Medical Image Analysis* 68 (2021): 101909.

6 Çiçek, Özgün, et al. "3D U-Net: learning dense volumetric segmentation from sparse annotation." *International conference on medical image computing and computer-assisted intervention*. Springer, Cham, 2016.

7 Harrison, Adam P., et al. "Progressive and multi-path holistically nested neural networks for pathological lung segmentation from CT images." *International conference on medical image computing and computer-assisted intervention*. Springer, Cham, 2017.

8. Lee C, et al. "Deeply-supervised nets." Artificial intelligence and statistics. Proceedings of Machine Learning Research, 2015.

9 Yousefi S, Sokooti H, Elmahdy MS, et al. Esophageal gross tumor volume segmentation using a 3d convolutional neural network. International conference on medical image computing and computer-assisted intervention. Springer. 2018. pp. 343-351.

Supplemental Tables

TABLE A1 | Subject and imaging characteristics in the multi-user study.

Characteristics	Multiuser study Institutions 2-4 (n = 20)
Sex	…
Male	18 (90%)
Female	2 (10%)
Diagnostic age	69 [61-79]
Clinical T stage	…
cT2	4 (20%)
cT3	10 (50%)
cT4	6 (30%)
Tumor location	…
Cervical	2 (10%)
Upper third	3 (34%)
Middle third	12 (60%)
Lower third	11 (55%)
BMI	…
< 18.5	7 (35%)
18.5 – 23.9	11 (55%)
> 24	2 (10%)
Imaging available	…
pCT	20 (100%)
PET/CT	0 (0%)

Note: patients may have tumors located across multiple esophagus regions, hence, total numbers summed at various tumor locations for the entire and sub-institution cohorts are greater than the corresponding total patient numbers. Age is presented as median and [interquartile range]. pCT = treatment planning CT.

TABLE A2 | Contour accuracy comparison to pCT-based DenseUNet method (9) in external multi-institutional testing.

Metrics	Our pCT deep model	DenseUNet model	*p* value
DSC: mean (95% CI)	0.80 (0.78, 0.81)	0.75 (0.73, 0.77)	<0.001
HD95 (mm): mean (95% CI)	11.8 (10.1, 13.4)	15.5 (13.0, 18.1)	<0.001
ASD (mm): mean (95% CI)	2.8 (2.4, 3.2)	4.3 (3.5, 5.2)	<0.001

Note: Wilcoxon matched-pairs signed rank test was used to compare the performance of our developed model and the DenseUNet model (9). pCT = treatment planning CT. DSC = Dice similarity coefficient, HD95 = 95% Hausdorff distance, ASD = average surface distance. CI = confidence internal.

TABLE A3 | Experts' assessment of degrees of manual revision in the subgroup analysis stratified by clinical T stages and tumor locations.

	Institution 2-4 (external multi-institutional testing)				
Input images	pCT				
Degree of manual revision	no revision (n=220)	>0-10% (n=49)	>10%-30% (n=42)	>30%-60% (n=10)	unacceptable (n=33)
Clinical T stage					
cT2 (n=74)	38 (52%)	4 (5%)	6 (8%)	3 (4%)	23 (31%)
cT3 (n=177)	125 (70%)	26 (15%)	17 (10%)	4 (2%)	5 (3%)
cT4 (n=103)	57 (55%)	19 (18%)	19 (18%)	3 (3%)	5 (5%)
Tumor location					
Cervical (n=60)	37 (62%)	7 (12%)	10 (17%)	2 (3%)	4 (6%)
Upper third (n=143)	82 (57%)	21 (15%)	25 (17%)	4 (3%)	11 (8%)
Middle third (n=178)	107 (60%)	31 (18%)	18 (10%)	6 (3%)	16 (9%)
Lower third (n=70)	45 (64%)	5 (7%)	10 (14%)	4 (6%)	6 (9%)

Note: the χ^2 test was used to compare the difference in degrees of manual revision between subgroups. A significantly higher percentage of patients required minor (0-30% slice revision) or no revision in advanced cT3 and cT4 stages as compared to that in early cT2 stage (95% and 91% vs 65%, $p<0.01$). cT2 = clinical T-stage 2, cT3 = clinical T-stage 3, and cT4 = clinical T-stage 4. pCT = treatment planning CT.

TABLE A4 | Contouring accuracy comparison of four radiation oncologists for their 1st time manual delineation.

Evaluation metrics	R1	R2	R3	R4	Deep model
DSC	…	…	…	…	…
mean (95% CI)	0.82 (0.79, 0.85)	0.83 (0.80, 0.85)	0.79 (0.76, 0.83)	0.82 (0.79, 0.85)	0.82 (0.80, 0.84)
p value	0.98	0.65	0.24	0.98	--
HD95 (mm)	…	…	…	…	…
mean (95% CI)	9.3 (6.9, 11.7)	8.3 (6.8, 9.8)	12.0 (7.7, 16.3)	9.1 (6.5, 11.7)	7.9 (5.7, 10.0)
p value	0.15	0.73	**0.01**	0.56	--
ASD (mm)	…	…	…	…	…
mean (95% CI)	1.9 (1.4, 2.4)	1.8 (1.5, 2.1)	2.6 (1.6, 3.5)	2.0 (1.4, 2.5)	2.0 (1.6, 2.4)
p value	0.57	0.29	0.62	0.47	--

Note: R1 to R4 represent the 4 radiation oncologists involved in the multiuser evaluation. The Wilcoxon matched-pairs signed rank test was used to compare the performance of our developed model and the four radiation oncologists. CI = confidence internal, DSC = Dice similarity coefficient, HD95 = 95% Hausdorff distance, ASD = average surface distance.

TABLE A5 | Contouring accuracy comparison of four radiation oncologists between their 1st time manual delineation and 2nd time deep learning assistant delineation in terms of DSC and HD95.

Radiation oncologists	DSC mean (95% CI)	*p* value	HD95 (mm) mean (95% CI)	*p* value
R1		0.14		0.18
1st time manual contour	0.82 (0.79, 0.85)		9.3 (6.9, 11.7)	
2nd time assisted contour	0.84 (0.82, 0.86)		7.5 (5.3, 9.6)	
R2		**<0.01**		**0.03**
1st time manual contour	0.83 (0.80, 0.85)		8.3 (6.8, 9.8)	
2nd time assisted contour	0.87 (0.85, 0.88)		6.2 (4.6, 7.7)	
R3		**0.01**		**<0.01**
1st time manual contour	0.79 (0.76, 0.83)		12.0 (7.7, 16.3)	
2nd time assisted contour	0.83 (0.80, 0.85)		7.8 (5.4, 10.1)	
R4		0.44		0.17
1st time manual contour	0.82 (0.79, 0.85)		9.1 (6.5, 11.7)	
1st time manual contour	0.83 (0.81, 0.86)		7.4 (5.3, 9.5)	
Average of R1 to R4		**<0.001**		**<0.001**
1st time manual contour	0.82 (0.80, 0.83)		9.7 (8.3, 11.1)	
1st time manual contour	0.84 (0.83, 0.85)		7.2 (6.2, 8.2)	

R1 to R4 represent the 4 radiation oncologists involved in the multiuser evaluation. The Wilcoxon matched-pairs signed rank test was used to compare the radiation oncologist's performance of the 1st time manual contouring and 2nd time assisted contouring. DSC = Dice similarity coefficient, HD95 = 95% Hausdorff distance.

Supplemental Figure

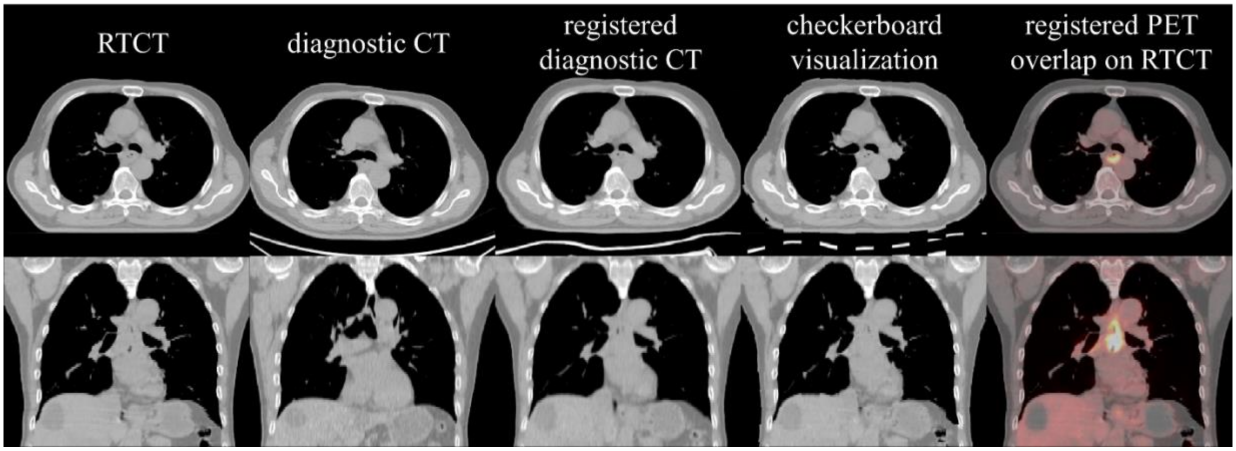

FIGURE A1 | An example of the deformable registration results for a patient in axial and coronal views. From left to right are the pCT image; the PET-CT image (accompanying PET) before and after the registration, respectively; a checkerboard visualization of the pCT and registered diagnostic CT images; and finally, the overlapped PET image, transformed using the diagnostic CT deformation field, on top of the pCT.

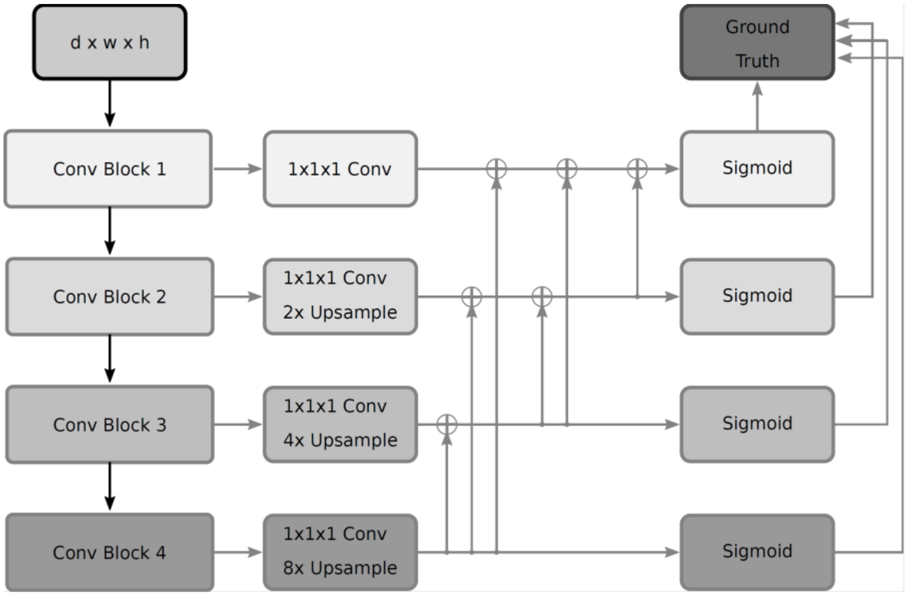

FIGURE A2 | Illustration of the 3D progressive semantically-nested network (PSNN) model, which employs deep supervision at different scales within a parameter-less high-to-low level image segmentation decoder. Four 3D convolutional blocks are applied. The first two and last two blocks are composed of two and three 3 ×3 ×3 convolutional + BN + ReLU layers, respectively.

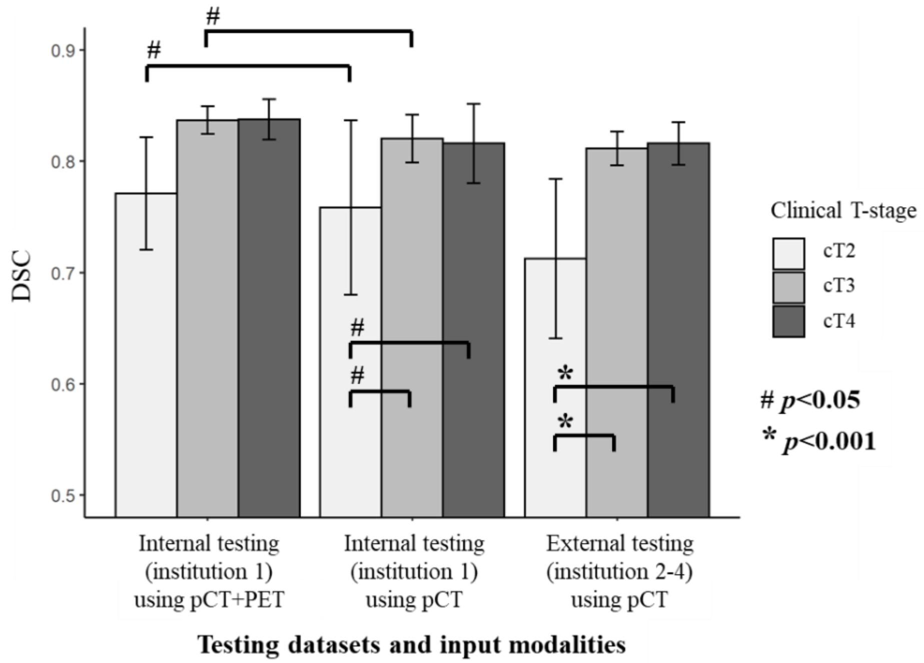

FIGURE A3 | Subgroup analysis of the deep model performance stratified by the clinical T-stage in both internal and external testing datasets. Manning-Whitney U test was used to compare the DSC of different clinical T-stage patients in each testing dataset and input modality. The Wilcoxon matched-pairs signed rank test was used to compare the DSC between the model using pCT and that using the pCT+PET in internal testing. Using pCT as input, DSC of cT2 patients yielded significantly lower values as compared to that of cT3 and cT4 patients in both internal (mean DSC: 0.76 vs 0.82, 0.82, $p<0.05$) and external (mean DSC: 0.71 vs 0.81, 0.82, $p<0.001$) evaluation. This phenomenon was less prominent after adding PET as additional input. In the internal evaluation, using pCT+PET as input, DSC of patients of cT2 and cT3 had significantly improved performance ($p<0.05$) over those using pCT alone as input. cT2 = clinical T-stage 2, cT3 = clinical T-stage 3, and cT4 = clinical T-stage 4. pCT = treatment planning CT, DSC = Dice coefficient similarity.

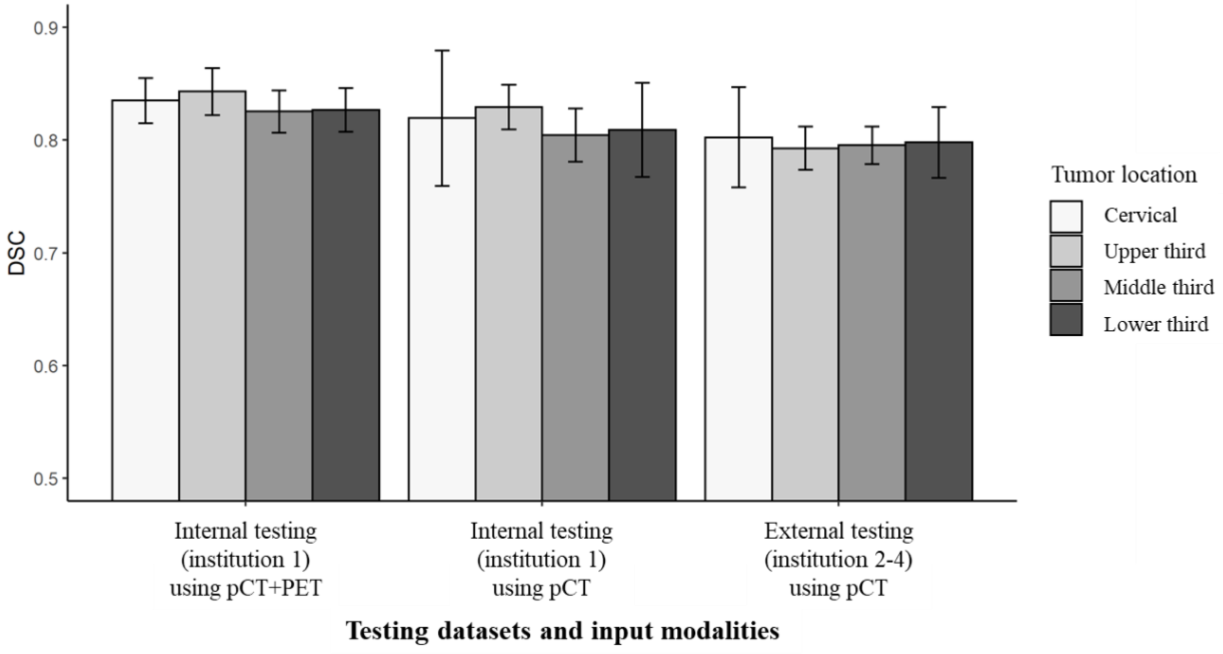

FIGURE A4 | Subgroup analysis of the deep model performance stratified by the tumor location in both internal and external testing datasets. pCT = planning CT, DSC = Dice coefficient similarity. Multiple linear regression with stepwise model selection was used to compare the DSC at different tumor locations, since a large tumor may locate across multiple esophagus regions. No significant differences were observed for the deep model performance at four tumor locations in both internal and external testing datasets or using pCT and pCT+PET as input images. This demonstrated the robustness of our deep model at different tumor locations. cT2 = clinical T-stage 2, cT3 = clinical T-stage 3, and cT4 = clinical T-stage 4. pCT = treatment planning CT, DSC = Dice coefficient similarity.
